# Medical Education

**Published:** 1859-07

**Authors:** Joseph P. Logan


### MEDICAL EDUCATION.

We regard as very just and well-timed the following observations of Prof. Joseph P. Logan, which we find in his recent excellent address, at the Atlanta Medical College:

"It has even become a favorite object with some who claim a place in our ranks, to degrade the American medical profession, in the estimation of the world, and to decry what is called in derision the 'American System of Medical Education.' Now, gentlemen, I am not here upon this occasion as the special champion of the institution which you have honored with your presence, or of any other medical college. Nor am I here to defend and endorse *as perfect*, the plan of educating medical men in the United States, but I am free to confess that I am heartily sick of, and prepared to denounce, as false, the stereotyped cry of the inferiority of the medical profession as compared with that of Europe. It is high time, in my judgment, that the supercilious and superficial traducers of American medicine, in or out of the profession, should be met by the facts. I would not wish to discourage any effort upon your part to master all the truths connected with our science, but rather to encourage you to the most indefatigable efforts to fit yourselves for the faithful discharge of the solemn obligations resting upon all those who assume the responsible position of physician. But I consider myself fully authorized in asserting that the regular medical men of the United States are offering an amount of science and skill far exceeding their appreciation upon the part of the public, and furnishing a standard of qualification far higher than the people deserve, and fully equal to the demand —the highest order of science and skill in every department of our comprehensive profession, being readily and conveniently commanded by every community that has any proper conception of the remuneration that should be awarded to the self-sacrifice and toil, which you may be assured, is ever inseparably connected with eminence in medicine."

"Indeed, we learn from a late reviewer of medical education in Great Britain (which may be taken as a fair representative of Europe), as exhibited in an elaborate article in the *Westminster Review*, that there is great injustice in supposing that the American medical profession can be benefitted by any usage or example supplied by the profession in Great Britain; but, on the contrary, it is shown that there is no advantage in the comparison to the European medical man, in either mind or attainments."

"I cannot, therefore, consider it inappropriate to the subject or the occasion, to declare, upon the highest authority, the testimony being based upon a practical familiarity with Europe—and after a somewhat laborious investigaton of this question, that the American pupils in attendance upon foreign schools of medicine, are in no ordinary degree superior to the classes with which they are associated, and that the mass of physicians in Europe are in no respect superior to the mass in the United States, but, on the contrary, in their treatment of disease, it is fearlessly repeated that they are decidedly inferior."*

